# New, Optimized Skin Calorimeter Version for Measuring Thermal Responses of Localized Skin Areas during Physical Activity

**DOI:** 10.3390/s24185927

**Published:** 2024-09-12

**Authors:** Miriam Rodríguez de Rivera, Pedro Jesús Rodríguez de Rivera

**Affiliations:** 1 Cardiology Service, Hospital Universitario Marqués de Valdecilla, 39008 Santander, Spain; miriam.mrdrs@gmail.com; 2 Department of Physics, University of Las Palmas de Gran Canaria, 35001 Las Palmas de Gran Canaria, Spain

**Keywords:** conduction calorimetry, direct calorimetry, skin heat flux, skin calorimeter, skin thermal properties, sports medicine sensors

## Abstract

We present an optimized version of the skin calorimeter for measuring localized skin thermal responses during physical activity. Enhancements include a new holding system, more sensitive thermopiles, and an upgraded spiked heat sink for improved efficiency. In addition, we used a new, improved calorimetric model that takes into account all the variables that influence the measurement process. Resolution in power measurement is 1 mW. Performance tests under air currents and movement disturbances showed that the device maintains high accuracy; the deviation produced by these significant disturbances is less than 5%. Human subject tests, both at rest and during exercise, confirmed its ability to accurately measure localized skin heat flux, heat capacity, and thermal resistance (less than 5% uncertainty). These findings highlight the calorimeter’s potential for applications in sports medicine and physiological studies.

## 1. Introduction

The localized measurement of skin thermal response is essential for understanding the thermal regulation of the human body and for the study of several physiological and pathological conditions. Heat flux, thermal resistance, and heat capacity significantly influence the thermal behavior and temperature of the skin [1]. At rest, the human body dissipates between 70 and 80 W. During physical activity, dissipation can increase up to 1500 W. Most of this heat is dissipated by convection, radiation, and evaporation [2,3]. Localized measurements of skin heat flux are typically performed using heat flux sensors. However, these sensors disturb the heat flow in the measurement area, leading to results that depend on the sensor type and on the ambient conditions. Several studies have investigated skin heat flux at rest [4], as a function of clothing [5], while heating or cooling the subject [6,7], during different physical activities [8,9], or under extreme environmental conditions [10]. In a recent work, we studied the lack of standardization in skin heat flux measurements, and we proposed a normalization method [11].

On the other hand, indirect calorimetry is used to assess metabolic rates and energy expenditure and is considered today the gold standard, although it does not provide localized information [12]. From heat flow and its variations with temperature, it is possible to measure the heat capacity and thermal resistance of the skin in vivo. Sensors for measuring these quantities typically include integrated heating elements and temperature sensors [13].

Thermal resistance relates the power transmitted through the skin with the temperature change associated. This magnitude is macroscopic, and it is characteristic of the skin area analyzed. Thermal resistance depends on tissue composition, water content, blood flow, perfusion, etc. Several instruments and methods have been developed, such as the Hematron, which consists of a disc with a central heater and temperature sensors in the perimeter [14]; the method of Wang et al., based on heat flux sensors [10]; the ring flat conductivity meter [15]; the ultrathin conformal array [16]; the flexible 3ω sensor [17]; and the guarded thermistor probe of Okabe [18,19]. These measurements have been useful for evaluating vasoconstriction and vasodilatation, to study the effects of compression stockings, and even in cancer studies. In a recent work, we studied the relation between the thermal resistance measured by several authors and the heat-affected zone, which is one of the major difficulties in this field [20].

Regarding heat capacity, differential scanning calorimetry (DSC) is commonly used to measure heat capacity in vitro [21]. Heat capacity depends on the tissue composition and mainly on its water content. The field of in vivo heat capacity measurement did not develop significantly until 2009, when the 3ω method was proposed to measure the thermal properties of the skin [22]. Since then, the most notable instrumentation in the field are Webb RC’s ultrathin conformal arrays of sensors and actuators [16,23], Li Gao’s epidermal photonic devices [24], and Limei Tian’s flexible and stretchable 3ω sensors [17]. In a recent work, we studied the coherence between all these results and its dependence with thermal penetration depth and water content [25]. The applications of these measurements are still under development, but some of these methods have already been used to monitor small skin lesions [26].

Parallel to the development of these instruments, our team has developed a calorimetric sensor, also known as a skin calorimeter. This device was designed to measure the heat flux, thermal resistance, and heat capacity of a 2 × 2 cm^2^ square skin region. After several experimental measurements, it has been observed that one of the major limitations of this type of instrument is measuring during physical activity. Actually, there is no technology to measure the thermal behavior of localized skin areas during physical activity with enough resolution. In this work, we present a new version of the skin calorimeter, optimized for measurements during physical exercise. This instrument is a new, valuable tool in the field of physical exercise sensors, enabling precise monitoring of heat flux in localized moving parts of the human body, useful in the field of in vivo localized measurements. In addition to the improved design, a more efficient calorimetric model has been employed, allowing continuous and immediate measurement of fast thermal transients. First, the device characteristics and the calorimetric model are presented. Then, the operating range, the sensor’s capacities, and its limitations are explained. Finally, an example of experimental measurement during physical exercise is presented.

## 2. Materials and Methods

### 2.1. Skin Calorimeter

**Figure 1:**
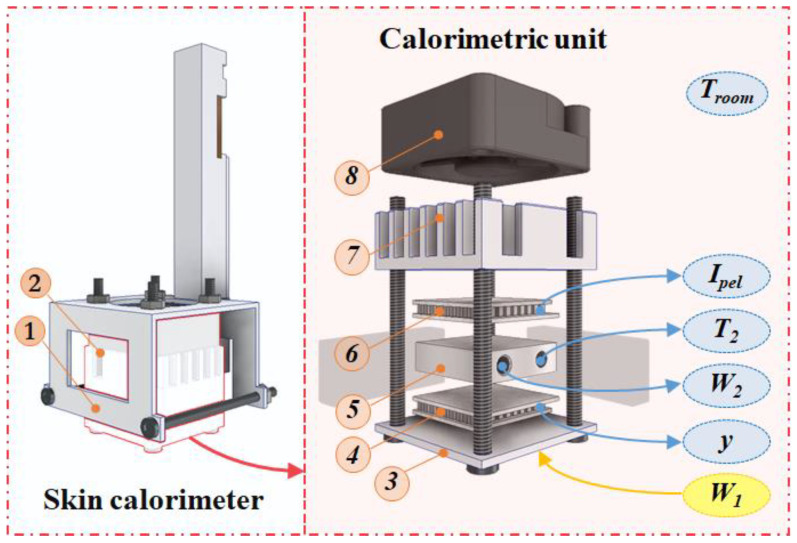
New skin calorimeter exploded view: (1) holding system, (2) calorimetric unit, (3) measurement plate, (4) measurement thermopile, (5) thermostat, (6) cooling thermopile, (7) heat sink, (8) fan. The main measured variables are indicated in the figure and explained in the text.

The new skin calorimeter design retains the same operating principle as its predecessors but includes several improvements. A new holding system has been introduced to facilitate easy application on the skin (part 1 in Figure 1). The calorimetric unit remains similar to previous versions (part 2 in Figure 1). 

A measuring thermopile (part 4 in Figure 1) generates the calorimetric signal *y*, which is proportional to the temperature difference between the measuring plate (part 3 in Figure 1), responsible for transmitting the power *W_1_* dissipated by the human body or a calibration resistor, and the thermostat (part 5 in Figure 1) with a temperature *T_2_*, resulting from the *W_2_* power dissipated inside it (PID control is used). The cooling system consists of a cooling thermopile (part 6 in Figure 1), a heat sink (part 7 in Figure 1), and a fan (part 8 in Figure 1). The fan (MF20C05, SEPA Europe, Eschbach, Germany) operates at 5 V supply. The cooling thermopile supply is variable and defined by its current (*I_pel_*). The aluminum heat sink has been upgraded from a simple extruded heat sink to a machined spiked heat sink, enhancing its efficiency. Both the cooling and measuring thermopiles have been replaced with more sensitive modules (ET2065F2A131211W2.25, Laird Technologies, Chesterfield, MO, USA), increasing the device’s overall sensitivity. This version of the calorimeter can operate from 10 °C below the room temperature (*T_room_*).

The mechanical enhancement includes threading the screws into the measuring plate, making this joint function more like a recessed joint rather than a hinge. This modification reduces the deformation of the measuring plate, thereby improving the device’s sensitivity and response time by minimizing the gap between elements. During assembly, thermal paste is applied between all components to ensure an optimal thermal contact. In addition, the thermostat and the measuring and cooling thermopiles are insulated on the sides with expanded polystyrene and protected from radiation by a layer of reflective aluminum foil.

With these improvements, the calorimeter can operate over a wide range of ambient temperatures (18–32 °C) and thermostat temperatures (15–40 °C) without temperature control saturation. The calorimeter design facilitates heat transfer between the sample and the thermostat and reduces external disturbances, enabling it to operate in motion or with slight air currents, as we will see later.

### 2.2. Calorimetric Model

Originally, the calorimetric model consisted of a MIMO (multiple input–multiple output) system with two inputs and two outputs. The inputs are the power dissipated by the skin (*W_1_*) and the power dissipated in the thermostat (*W_2_*). The outputs are the thermostat temperature (*T_2_*) and the calorimetric signal (*y*). In this new device, we incorporated two new inputs into the model to accurately represent the behavior of the calorimeter: the ambient temperature (*T_room_*) and the current of the cooling thermopile (*I_pel_*). Note that these two variables are not usually taken into account in calorimeters because they usually remain constant during calorimetric measurements. In calorimetry, the energy measurements are taken considering the initial state as a reference [27]. However, in physical activities, the local room temperature (*T_room_*) around the calorimeter shows alterations during the measurement. These alterations are influenced by the cooling thermopile (*I_pel_*). To simplify the calorimeter operation, the cooling thermopile operates in a range of intensities in which its behavior can be considered linear. Then, the system has four inputs and two outputs.

The calorimetric model is based on a physical image model that considers two bodies: the sample and the calorimeter itself. Each body is represented by its temperature (*T_1_* and *T_2_*) and heat capacity (*C_1_* and *C_2_*). The bodies are connected by thermal couplings: the thermal conductance between the sample and the environment is *P_1_*, the thermal conductance between the two bodies is *P_12_*, and the thermal conductance between the second body and the cold focus is *P_2_*. Considering the power developed in each body, the equations of the model are as follows (1):

(1) $$\begin{array}{l} W_{1}=C_{1}\left(\frac{1}{k}\frac{dy}{dt}+\frac{dT_{2}}{dt}\right)+P_{1}\left(T_{2}-T_{room}-\beta I_{pel}+\frac{1}{k}y\right)+P_{12}\left(\frac{1}{k}y\right)\\ W_{2}=C_{2}\left(\frac{dT_{2}}{dt}\right)+P_{2}\left(T_{2}-T_{room}\frac{}{}\alpha I_{pel}\right)-P_{12}\left(\frac{1}{k}y\right) \end{array}$$

where *k* is the Seebeck coefficient of the measurement thermopile. Parameter *α* represents how the cooling thermopile current (*I_pel_*) affects the cold focus temperature, and *β* represents how *I_pel_* affects the ambient temperature around the calorimeter.

The behavior of the calorimeter is represented by these equations, and all the variables are measurable at each instant with a sampling period of 0.5 s. The parameters to be identified are the heat capacities *C_1_* and *C_2_*; the thermal conductances *P_1_*, *P_2_*, and *P_12_*; the Seebeck coefficient *k*; and the parameters *α* and *β*. All the parameters mentioned depend on the calorimeter’s parts and, hence, are constant, except for two: *C_1_* and *P_1_*, which depend on the sample under the calorimeter.

### 2.3. Calibration

**Figure 2:**
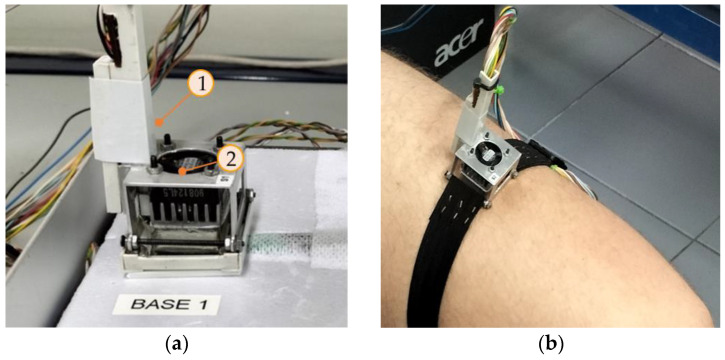
New skin calorimeter (**a**) placed in its calibration base and (**b**) placed on the thigh of a human subject. (1) holding system, (2) calorimetric unit.

To calibrate the calorimeters, we used a calibration base that consists of an EPS block with a little aluminum block that contains a thermostat. This allows the calibration of the device. Figure 2 shows the calorimeter placed on its calibration base (a) and the calorimeter applied on the thigh of a human subject (b). In this paper, we present the calibration of two calorimeters, designated as *S_1_* and *S_2_*.

**Figure 3:**
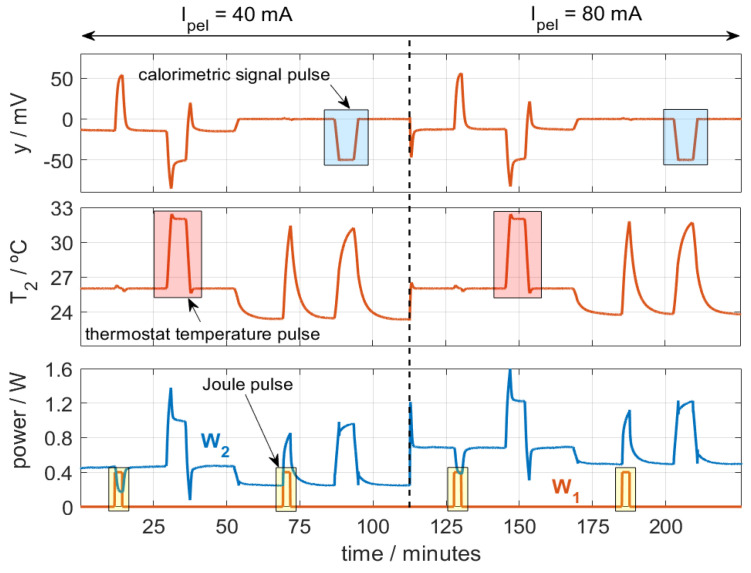
Calibration measurement with temperature (*T_2_*_,_ from 26 to 32 °C), calorimetric signal (*y*, from 0 to −50 mV), and Joule pulses (*W_1_*_,_ from 0 to 0.4 W) for different *I_pel_* values. *T_room_* = 23 °C.

We performed several calibrations at various thermostat temperatures (*T_2_*) and cooling thermopile currents (*I_pel_*). Both the thermostat temperature and the calorimetric signal were regulated using a PID controller, with a sampling period of 0.5 s. The purpose of these calibrations is to accurately represent the behavior of the calorimeter over its entire operating range. Figure 3 shows one of the calibration measurements performed. 

In the figure, three types of pulses are programmed: temperature pulses (*T_2_*, from 26 to 32 °C), calorimetric signal pulses (*y*, 0 to −50 mV), and Joule pulses (*W_1_*_,_ from 0 to 0.4 W). The ambient temperature was *T_room_* = 23 °C. Figure 3 represents the signals for calorimeter *S_1_*, which behaves similarly to calorimeter *S_2_*. To identify the model parameters, we use an iterative method used in previous works [28] that reconstructs the outputs of the model (calorimetric signal and thermostat temperature, *T_2_* and *y*) from the inputs: the powers *W_1_* and *W_2_*, the ambient temperature *T_room_*, and the cooling thermopile current *I_pel_*. To evaluate the accuracy of the model parameters obtained, we used an error criterion that consists of the root mean square error (RMSE) between the calculated signals (*y_c_* and *T_2,c_*) and the original ones (*y_0_* and *T*_2,0_). With *n* being the number of points, the error criterion is the following (2):

(2) $$\varepsilon =\frac{1}{n}\sqrt{\sum_{i=1}^{n}{\left(y_{0}(i)-y_{c}(i)\right)}^{2}}+\frac{1}{n}\sqrt{\sum_{i=1}^{n}{\left(T_{2,0}(i)-T_{2,c}(i)\right)}^{2}}\;$$

Table 1 shows the mean values of the parameters obtained in the calibration process for both calorimeters *S_1_* and *S_2_*. The mean RMSE of the fitting is 0.15 mW, 6.4 μV, and 0.9 mK for *W_1_*, *y*, and *T_2_*, respectively.

## 3. Results and Discussion

We have divided the results and discussion section into four parts. First, an experimental study is performed on two types of disturbances that affect the normal operation of skin calorimeters. The first involves air flows in the room caused by open windows, doors, or air-conditioning systems. The second disturbance is the sensor’s movement when attached to a part of the body that is exercising or moving. Finally, some measurements on the human body at rest and during exercise will be presented.

### 3.1. Experimental Performance Evaluation: Disturbances Caused by Air Currents

To simulate the disturbance caused by the air flow in the room where measurements are performed, a small air blower is directed over the calorimeters placed on the calibration base. Using a small air blower at a distance of 1–2 m can simulate a range of air velocities common in various scenarios of interest. Air blowers typically produce air speeds of 8–10 m/s, which are comparable to the velocities of air conditioning units (2–5 m/s), fans (2–8 m/s depending on the type), and natural air currents entering through a window (1–10 m/s). Therefore, an air blower can be a practical tool for experimental setups requiring varied airflow conditions. These speeds are higher than typical speeds in room measurements but are achievable in some experiments of high physical exercise performance. The blower used is an old AEPI hair dryer (220 V 450 W) running in cold mode. The air speed in the area of the sensors was 8 m/s.

**Figure 4:**
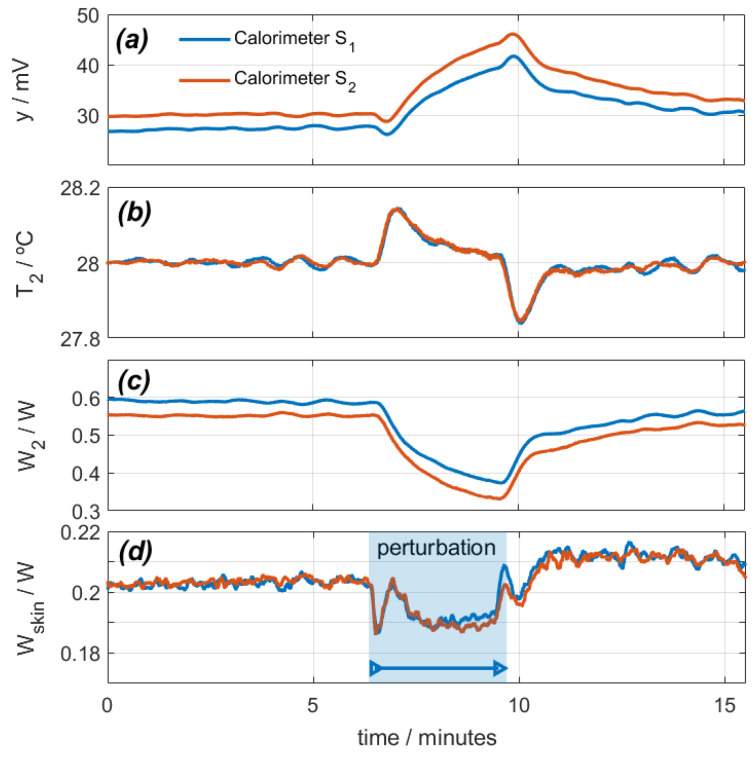
Effect of a disturbance caused by a directed air blower on calorimeters *S_1_* (blue) and *S_2_* (red): (**a**) calorimetric signals, (**b**) thermostat temperatures, (**c**) thermostat power, (**d**) calculated calibration base dissipated power (real power is 0.2 W).

With the calorimeters placed on their calibration base, a measurement was programmed in which the calibration resistor dissipated a constant power of 0.2 W, and the thermostat temperature was programmed for a constant value of 28 °C. The ambient temperature was 25.6 °C, and the cooling supply was 0.1 A. Figure 4 shows the calorimetric signal, the thermostat temperature, the power *W_2_*, and the calculated power *W_1_*. In this experimental measurement, the air impacts on the heat sink and fans of each calorimeter. For this reason, the hot air from these fans returns to the calorimeters, increasing the temperature around them. This results in an increase of the calorimetric signal (Figure 4a), a disturbance of the thermostat temperature (Figure 4b), and a decrease of the thermostat power (Figure 4c). Using the procedure described (Equation (1)), we calculate the power *W_1_*, as shown in Figure 4d. As a conclusion, we can state that this perturbation produces a deviation in the calculation of *W_1_* by 10 mW (5% of the real power).

### 3.2. Experimental Performance Evaluation: Disturbances Caused by Calorimeter Movements

**Figure 5:**
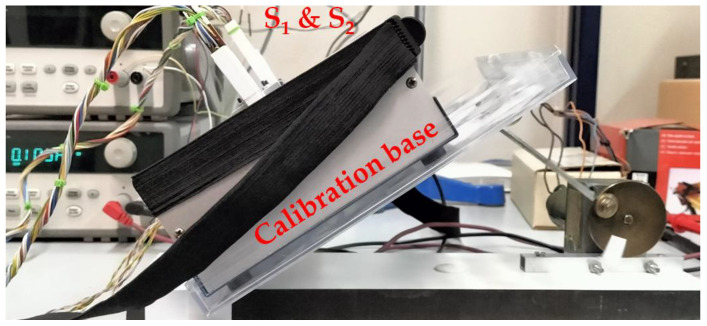
Experimental setup for studying the movement effect on the calorimeters.

When a subject is exercising and calorimeters are attached to the thigh (as in Figure 2b), the devices will move. To reproduce this movement, we constructed an oscillating system driven by a small direct current motor. We programmed oscillations at 112 rpm. The oscillations in normal pedaling on a bicycle range between 60 and 100 rpm. The calibration base and the calorimeters are placed on the oscillating system, as seen in Figure 5.

**Figure 6:**
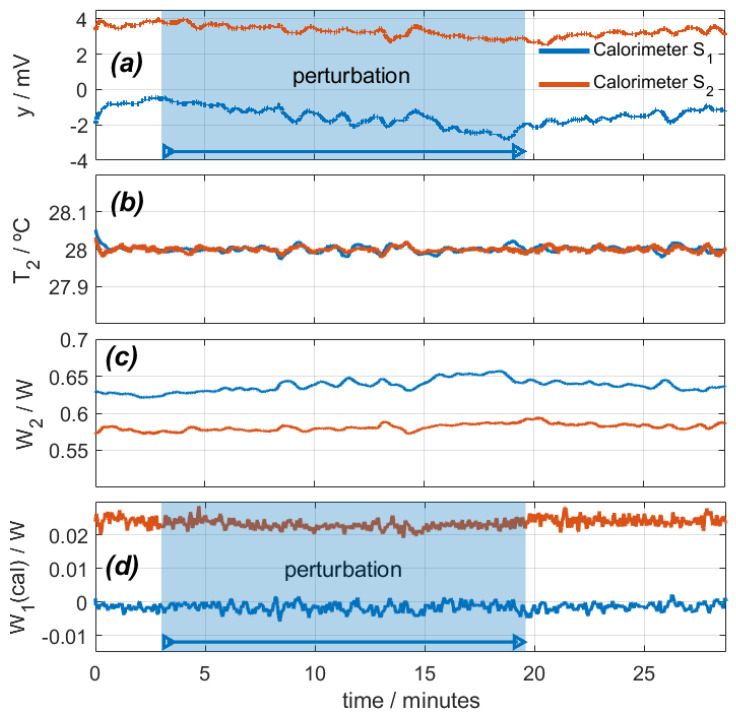
Study of the effect of sensor movement on an oscillating system (112 rpm): (**a**) calorimetric signals, (**b**) thermostat temperatures, (**c**) thermostat power, (**d**) calculated calibration base dissipated power (real power is 0 W for calorimeter *S_1_* and 0.025 W for calorimeter *S_2_*). *T_room_* = 27.1 °C; *I_pel_* = 0.1 A.

In this case, the calibration resistor dissipates zero power in calorimeter *S_1_* and 25 mW in calorimeter *S_2_*. Figure 6 shows the calorimetric signal, the temperature and power of the thermostat, and the calculated *W_1_* power. The disturbance lasts for 16 min.

When the disturbance begins, the calorimetric signal decreases due to cooling, the low-frequency oscillations slightly increase, and the high-frequency oscillations increase from ±2 mW to ±3 mW. *T_2_* is set at 28 °C, and *W_2_* is adjusted to maintain this temperature. As a result, the calculated power *W_1_* remains at zero for calorimeter *S_1_* and decreases by 1.5 mW for calorimeter *S_2_*, which is not significant considering the inherent noise.

### 3.3. Experimental Measurements on Human Body At Rest

**Figure 7:**
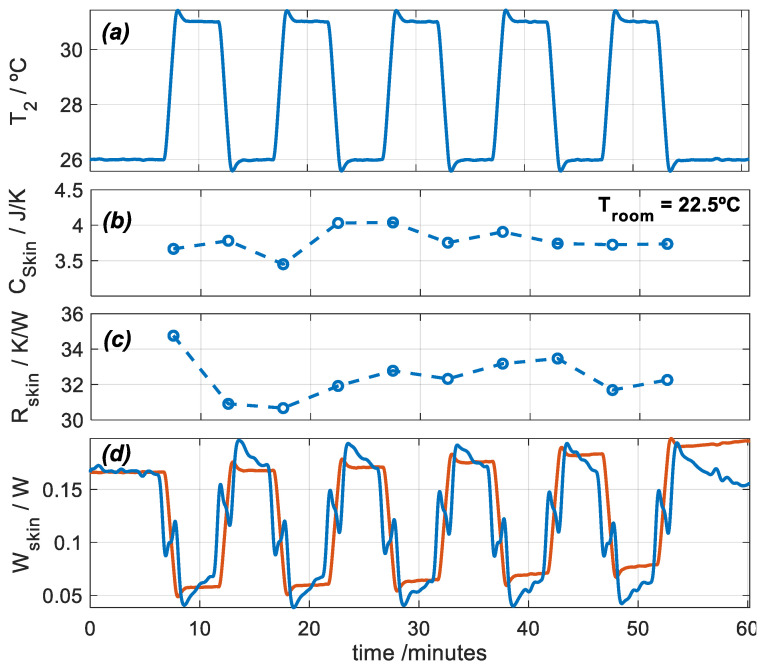
Experimental measurement performed on the thigh of a healthy 29-year-old male subject seated and at rest: (**a**) thermostat temperature *T_2_*, (**b**) skin heat capacity *C_skin_*, (**c**) skin thermal resistance *R_skin_*, and (**d**) skin heat flux *W_skin_* (blue: deconvolution, red: model, Equation (3)). *T_room_* = 22.5 °C, and RH = 61%.

In this section, we present a measurement with the subject at rest and seated. Using the calorimetric model (Equation (1)) and an iteration process similar to the one used in calibration, we are able to determine the heat flux, the heat capacity, and the thermal resistance of the skin. Measuring heat capacity and thermal resistance requires a thermal perturbation which, in our case, consists of a thermostat temperature pulse. In measurements at rest, this method takes 5–10 min and is accurate. With the calorimeter, we have performed interesting measurements, for example, to monitor the evolution of a second-degree burn [26]. Figure 7 shows an experimental measurement performed on the thigh of a healthy 29-year-old male subject (Figure 2). The subject was seated and at rest. To determine the thermal properties of the skin, thermostat temperature *T_2_* is programmed between 26 and 31 °C periodically. Figure 7 shows the thermostat temperature and the results of the calculation process: the heat capacity *C_skin_ = C_1_*, the thermal resistance *R_skin_* = 1/ *P_1_*, and the heat flux of the skin *W_skin_ = W_1_*.

To obtain these results, two factors were considered when measuring on skin:

- Heat flux can be determined by direct deconvolution by performing an inverse filtering of the first system equation of the calorimetric model (Equation (1)). This result is shown by the blue signal in Figure 7d. For this, mean values of *C_skin_* and *R_skin_*, shown in Figure 7b,c, were used. However, a simple model can represent skin heat flux, as given by Equation (3), which reproduces the inverse form of the thermostat temperature (red signal in Figure 7d). This method is valid only for rest measurements. For exercise measurements, we used direct deconvolution.

(3) $$W_{skin}(t)=W_{skin}(0)-\frac{T_{2}(t)-T_{2}(0)}{{P_{12}}^{-1}+R_{skin}}\;\;$$

- When the calorimeter is applied on the skin, the room temperature around the sensor increases. This rise in room temperature (∆*T_room_*) must be determined. This can be easily done using the second system equation of the calorimetric model (Equation (1)).

### 3.4. Experimental Measurements on Human Body During Exercise

**Figure 8:**
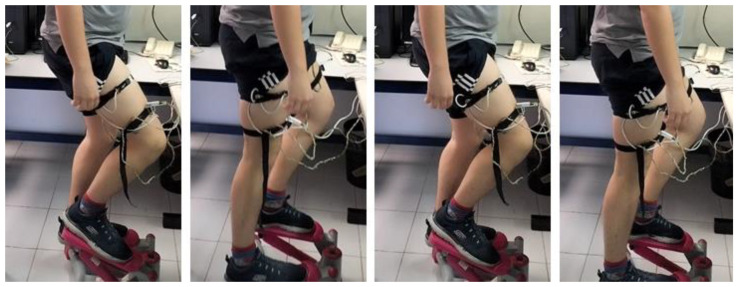
Healthy 29-year-old male subject performing physical exercise on a stair stepper.

In the case of physical activities, heat capacity and thermal resistance cannot be measured because the thermal responses of the human body interfere with the temperature pulses. To estimate *C_1_* and *P_1_*, we measured these parameters before and after physical activity (Figure 7b,c). Now, two experimental measurements were conducted to analyze the thermal responses of a healthy 29-year-old male subject engaged in physical exercise on a stair stepper (Figure 8).

**Figure 9:**
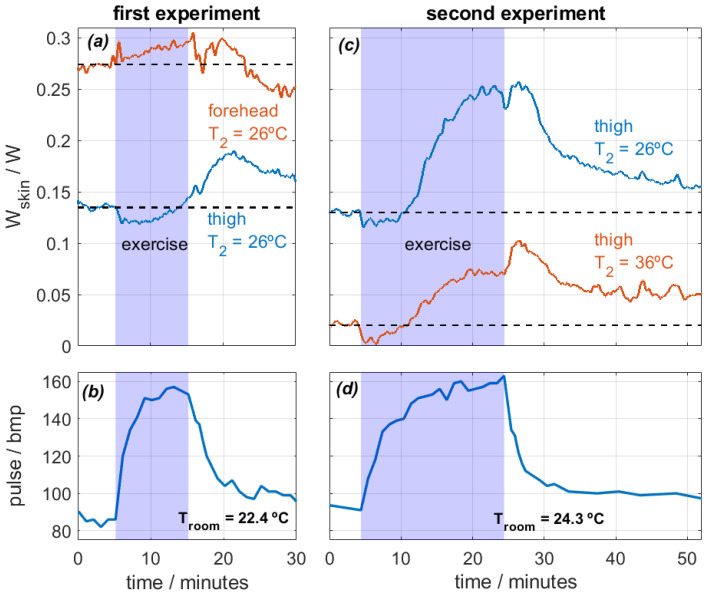
Experimental measurements performed on the forehead and thighs of a healthy 29-year-old male subject performing physical exercise on a stair stepper. First experiment (**a**,**b**): 10 min exercise, *T_room_* = 22.4 °C, measurements on the thigh and the forehead at *T_2_* = 26 °C. Second experiment (**c**,**d**): 20 min exercise, *T_room_* = 24.3 °C and HR = 58%, measurements on both thighs at *T_2_* = 26 and 36 °C. Heat flux *W_skin_* (**a**,**c**) and heart rate (**b**,**d**) are displayed.

The experiments focused on two specific body regions: the forehead and the thighs. The calorimeters measured continuously and were attached to the skin with the holding system shown in Figure 2. During the first experiment, the subject performed a 10-min exercise at a room temperature of *T_room_* = 22.4 °C. Measurements were taken from the thigh and the forehead, and the thermostat temperature was set at *T_2_* = 26 °C. In the second experiment, the subject exercised for 20 min at a room temperature of *T_room_* = 24.3 °C. This time, the measurements were recorded from both thighs at thermostat temperatures of *T_2_* = 26 °C and *T_2_* = 36 °C. The heat flux dissipated by the skin (*W_skin_*) and heart rate were monitored for both experiments. Figure 9 shows the results.

Regarding the first experiment, heat flux in the forehead was higher than in the thigh, which is expected [26]. Both values are obtained at the same thermostat temperature and are therefore comparable. When exercise begins, heat flux in the thigh decreases from 0.135 to 0.120 W (−11%) in the first six minutes. This slight reduction is caused by the initial blood flow redistribution. When exercise begins, the body prioritizes supplying blood to the active muscles. This causes a temporary blood flow reduction in the skin, which reduces the skin temperature and thus the heat flux [29].

As exercise continues, all parts of the body experience an increase in heat flux. The forehead, a zone far from the active muscles, experiences a slow heat flux increase over time from 0.274 W to 0.294 W (+7%) and starts to decrease when exercise ends. Note that the forehead does not experience the initial slight heat flux reduction. The thigh heat flux increases slowly over time, reaching a maximum value of 0.190 W (+41%). However, the maximum value is reached after exercise. This phenomenon illustrates the high thermal inertia of the human body; a stationary state is not reached during exercise. The heat flow starts to decrease at the end of the experiment, but it does not reach the initial steady state. For this reason, we prepared a second experiment with longer exercise.

In the second experiment, the heat flux evolution is similar in both thighs, but the values depend on the thermostat temperature *T_2_*, as expected. Heat flux decreases slightly in both cases for the first 6 min after the beginning of the exercise. In this case, the heat flux at *T_2_* = 26 °C increases up to 0.25 W (+92%), and the heat flux at *T_2_* = 35 °C increases up to 100 mW. After the exercise, the skin continues dissipating a high heat flux for another 5 min and then slowly decreases over time. Note that when the exercise is over, everything that was heated begins to cool down at different rates. In the forehead, sweating and evaporation were observed, and this probably caused the higher cooling. In the thighs, the cooling is slower. This is probably due to the lack of evaporation and the higher fat content in this zone of the human body, which leads to a higher heat capacity and thermal resistance and thus a higher time constant [26]. All these observations are coherent with the bibliography [6,7,8,9] and show the ability of the calorimeter to assess accurately the localized heat flux during physical activity.

## 4. Discussion

The experimental device was improved by increasing its sensitivity and making it more user-friendly. A precise methodology for measuring heat flux was also developed, which involves determining the thermal properties of the skin at the skin measurement zone beforehand. Heat flux measurements were conducted on an individual performing moderate physical exercise. Due to the high sensitivity of the instrument, it is possible to non-invasively detect transient phenomena that were previously only measurable through invasive methods, such as inserting a thermistor into the artery [20]. This instrument is a valuable tool in the field of physical exercise sensors, enabling precise monitoring of heat flux in localized moving parts of the human body, useful in the field of in vivo localized measurements. Table 2 compares the skin calorimeter (skin cal.) with a selection of commercial heat flux sensors widely used in research works (film and plate types). The table highlights the geometric characteristics (detection area and thickness), the thermal resistance, and the sensitivity of each sensor. Sensitivity (μV/Wm^−2^) is defined as the change in output signal (calorimetric signal) per change in heat flux, holding other variables constant. Sensitivity (mV/W) is also indicated as the ratio of the calorimetric signal variation to the variation of heat power. 

The skin calorimeter presents several strengths. It incorporates a programmable thermostat that increases its sensitivity compared to other sensors. Heat transmission occurs through conduction, and the thermopile is well-insulated, minimizing interference from radiation and convection. Additionally, the thermostat allows for measurements of heat flux and thermal properties of the skin. There are also significant opportunities for improvement. Further testing with a broader population, including various ages and genders, is necessary to validate its practical application in monitoring physical activity. Miniaturizing the calorimeter is another key opportunity, as this would allow integration into a multisensor device for wearable applications.

However, some challenges remain. Thermal measurements are inherently slow due to the high heat capacities of both the skin and the device. This makes it critical to manage measurement timing carefully to avoid overlapping thermal property readings with continuous heat flux measurements. Additionally, the cooling system requires manual selection of the cooling current for the thermopile to prevent thermostat saturation, which complicates operation and requires prior simulation. Without addressing these limitations, the device may become a bit cumbersome to use in dynamic or fast-paced applications.

## 5. Conclusions

This study presented an optimized version of the skin calorimeter for measuring localized skin thermal responses during physical activity. We conclude that the instrumentation enhancements and the new calorimetric model allow for high accuracy calibration, with an RMSE of less than 0.15 mW in power measurements. The new calorimeter showed acceptable deviations (5%) under air-current disturbances and stable power measurements during movement, but with increased oscillation (from ±2 mW to ±3 mW). These disturbances simulate measuring during physical activity.

During rest, measurements with thermostat temperature pulses (from 26 to 31 °C) allowed the accurate determination of the heat flux, the heat capacity, and the thermal resistance of the skin. As expected, the heat flux measured depends on the thermostat temperature set. The values obtained are consistent with previous works

Measurements of exercise showed that heat flux increases after exercise, as expected (between 41–90% at a thermostat temperature of 26 °C). However, this increase is not immediate; at the beginning of the exercise, we detected a slight heat flux reduction (8–11%) that lasted about 6 min. This is caused by an initial redistribution of blood flow in the active muscles. We also measured heat flux in the forehead, detecting a heat flux increase also, but with a different time constant. These dynamic behaviors are consistent with the literature.

This optimized skin calorimeter is an advance in direct calorimetry, providing accurate measurements of skin thermal responses under various conditions with significant potential for sport medicine and physiological research. The goal is to establish the skin calorimeter as a complementary and widely used tool for exercise monitoring. To validate its practical application, measurement campaigns are planned with subjects of various ages and sexes. Additionally, recent advances in its development will enable the miniaturization of the device, allowing its integration into a multisensor system for easy skin application.

## Figures and Tables

**Table 1:** Parameters obtained in the calibration for calorimeters *S_1_* and *S_2_*.

Parameter	Calorimeter *S_1_*	Calorimeter *S_2_*
*C_1_* ^(1)^	4.496 ± 0.032 J/K	4.042 ± 0.070 J/K
*C_2_*	3.456 ± 0.008 J/K	3.046 ± 0.032 J/K
*P_1_* ^(1)^	0.03566 ± 0.00062 W/K	0.03187 ± 0.00055 W/K
*P_2_*	0.06669 ± 0.00028 W/K	0.05641 ± 0.00023 W/K
*P_12_*	0.07111 ± 0.00360 W/K	0.06126 ± 0.00035 W/K
*k*	18.13 ± 0.62 mV/K	17.45 ± 0.68 mV/K
α	−80.79 ± 0.71 K/A	−84.55 ± 0.89 K/A
β	11.86 ± 0.29 K/A	18.63 ± 0.85 K/A

^(1)^ Parameters *C_1_* and *P_1_* shown in Table 1 correspond to the calibration base. When performing a measurement on the human body or over a sample, these two values will change.

**Table 2:** Comparison between the skin calorimeter and different commercial heat flux sensors.

Heat Flux	Sensing Area	Thickness	Sensitivity		Thermal Resistance	
Sensor	cm^2^	mm	μV/(Wm^−2^)	mVW^−1^	K/(Wm^−2^)	K/W
Film HFS-4 [30]	10.0	0.18	2.1	2.1	18 × 10^−4^	1.8
Film HFS-5 [31]	6.3	0.36	1.4	2.2	9 × 10^−4^	1.4
Film FHF05 [32]	1.0	0.40	1.0	10.0	11 × 10^−4^	11.0
Film FHF05 [32]	4.5	0.40	3.0	6.6	11 × 10^−4^	2.4
Plate HFP01 [32]	8.0	5.40	60.0	75.0	71 × 10^−4^	8.9
Skin cal. ^1^ [28]	4.0	2.20	58.4	146.0	36 × 10^−4^	9.0
Skin cal. ^1,2^	4.0	2.20	72.0	180.0	60 × 10^−4^	15.0

^1^ The measuring thermopile has an area of 13.2 × 13.2 mm and a thickness of 2.2 mm, but the calorimeter’s measurement plate has an area of 20 × 20 mm (4 cm^2^). ^2^ Device presented in this work.

## Data Availability

Data are contained within the article.
